# Development of a new serotyping ELISA for *Toxoplasma gondii* type II, type III and Africa 1 lineages using in silico peptide discovery methods, well categorized feline and human outbreak serum samples

**DOI:** 10.1186/s12879-022-07088-w

**Published:** 2022-01-31

**Authors:** Hüseyin Can, Ayşegül Aksoy Gökmen, Mert Döşkaya, Sedef Erkunt Alak, Aysu Değirmenci Döşkaya, Muhammet Karakavuk, Ahmet Efe Köseoğlu, Tuğba Karakavuk, Ceren Gül, Mervenur Güvendi, Aytül Gül, Adnan Yüksel Gürüz, Selçuk Kaya, Aurélien Mercier, Cemal Ün

**Affiliations:** 1 Molecular Biology Section, Department of Biology, Faculty of Science, Ege University, İzmir, Turkey; 2 Vaccine Development, Application and Research Center, Ege University, İzmir, Turkey; 3 Department of Microbiology, Faculty of Medicine, İzmir Katip Çelebi University, İzmir, Turkey; 4 Department of Parasitology, Faculty of Medicine, Ege University, İzmir, Turkey; 5 Ege University Ödemiş Technical Training College, İzmir, Turkey; 6 Graduate Faculty of Natural and Applied Science Biotechnology Program, Ege University, İzmir, Turkey; 7 Department of Bioengineering, Faculty of Engineering, Ege University, İzmir, Turkey; 8 Centre National de Référence (CNR) Toxoplasmose/Toxoplasma Biological Resource Center (BRC), Centre Hospitalier-Universitaire Dupuytren, Limoges, France; 9 INSERM, Université Limoges, CHU Limoges, IRD, U1094 Neuroépidémiologie Tropicale, Institut d’Epidémiologie et de Neurologie Tropicale, GEIST, Limoges, France

**Keywords:** Toxoplasmosis, Serotyping, Genotyping, GRA6, GRA7, Peptide ELISA

## Abstract

**Background:**

Discovery of new *Toxoplasma gondii* serotyping epitopes is important due to reports showing the influence of genotype on the severity of toxoplasmosis. In Turkey, genotypes belonging to type II, type III and Africa 1 lineages were mainly detected. The present study focused on to find out epitopes with high discriminative capacity to serotype these genotypes using well characterized strains isolated from Turkey.

**Methods:**

To meet this objective, GRA6 and GRA7 genes were sequenced from strains belonging to the type II, III and Africa 1 lineages, and B cell epitopes inside these sequences were predicted by Bcepred and additional docking analysis was performed with B cell receptor. Based on these analyses, 22 peptides harboring lineage specific epitopes were synthesized. Then, the serotyping potency of these peptides was tested using peptide ELISA and well categorized serum samples collected from stray cats infected with genotypes of the different lineages type II (n:9), III (n:1) and Africa 1 (n:1). As a result of peptide-ELISA, a serotyping schema was constructed with peptides that show high discriminative capacity and this assay was validated by sera collected from humans after an outbreak (n:30) and mother/newborn pair sera (n:3). Later, the validated serotyping schema was used to serotype a larger group of human (n:38) and cat (n:24) sera.

**Results:**

Among 22 peptides, GRA6II/c, GRA7III/d, and GRA6 Africa 1/b epitopes have shown discriminative capacity. During the validation of peptide-ELISA, the serotype of toxoplasmosis outbreak and mother/newborn cases were detected to be serotype II. Moreover, the analyses in a larger group showed that serotype II was prevalent in humans and stray cats.

**Conclusions:**

Overall, the results showed that the serotyping schema could be successfully used to serotype *T. gondii* infections caused by type II, III and Africa 1 genotype.

## Background

Toxoplasmosis is caused by an apicomplexan protozoan parasite known as *Toxoplasma gondii* that can infect all homeothermic animals (mammals and birds). Although toxoplasmosis is generally asymptomatic in healthy humans, clinical symptoms such as lymphadenopathy, chorioretinitis, and encephalitis can occur in immunocompromised patients. Also, transplacental transmission can cause hydrocephalus, blindness, mental defect or death of the fetus [1]. Approximately 30% of the human population are infected by various genotypes of *T. gondii* worldwide [2]. In Turkey, the prevalence of toxoplasmosis in domestic cats, as the definitive host of *T. gondii*, is between 30 and 40% [3–5].

Three major clonal lineages called as type I, II and III were well identified using several genetic markers including isoenzymes, single nucleotide polymorphisms and microsatellites [6]. In addition to these classical clonal lineages, a greater genetic diversity of *Toxoplasma* has been described associated with other divergent or recombinant lineages and occasionally some so-called atypical strains [6, 7]. Among the classical clonal lineages, type II is prevalent worldwide especially in Europe and North America. In Turkey, type II and III have been detected in domestic cats and wild animals [3, 4]. Africa 1 lineage has been detected in domestic cats and in two different human congenital toxoplasmosis cases [3, 8]. Concerning the pathological characteristics of these lineages, the classical types II and III are well known to be avirulent in laboratory mice and are weakly pathogenic in immunocompetent humans [6, 9, 10]. Africa 1 lineage has shown to cause congenital toxoplasmosis in humans and be virulent like type I in mouse models [8, 11–13]. Moreover, taking into account the studies dealing with the BrI lineage found in Brazil and which are very similar to the Africa I lineage (they belong to the same haplogroup 6 and are identical in microsatellite marker typing), the few studies in laboratory mice using this lineage show high virulence with most often 100% mortality of infected mice [9, 10, 14, 15]. A recent study performed in France detected cases of severe toxoplasmosis associated with strains belonging to the Africa 1 lineage in immunocompetent patients [16].

Currently, the investigation of the relationship between the genetic diversity of *Toxoplasma* strains and the pathogenesis of *T. gondii* infection is becoming a major issue due to the relationships described in the literature between strain genotypes and virulence observed both in mouse models and directly in humans [6, 17]. However, there are some limitations of classical genotyping methods (such as PCR-RFLP and microsatellite analyses) which need relatively high amount of parasite DNA to genotype *T. gondii* isolates in patient samples including blood, amniocentesis or vitreous fluid [1, 18, 19].

Serotyping based on the utilization of genotype specific peptides detected by advanced bioinformatics tools is a promising alternative method to genotype *T. gondii* strains. This method relies on detection of high-titer persistent IgG antibody in response to *T. gondii* proteins in humans and cats. To date, GRA3, GRA4, GRA5, GRA6, GRA7, NTPase I and III, SAG1, SAG2, SAG3, SAG4, BSR4, SRS2, ROP1, ROP5, ROP8, ROP16, ROP18, and ROP20 proteins have been studied by this approach [1, 18–22].

In Turkey, due to the detection of type II, III and Africa 1 lineages in domestic cats as well as in human congenital toxoplasmosis cases with Africa 1, the present study focused to discover *T. gondii* serotyping epitopes for type II, III and Africa 1 genotypes and to validate them in peptide-ELISA. To meet this objective, firstly GRA6 and GRA7 genes of *T. gondii* strains isolated from Turkey and genotyped as type II, III and Africa 1 by microsatellite method [3] were sequenced and translated to amino acid sequences by in silico methods. The sequences were then used to predict the genotype specific B cell epitopes by Bcepred tool taking into consideration the hydrophilicity, flexibility/mobility, accessibility, polarity, exposed surface and turns properties. Thereafter, docking analysis was performed for selected epitopes. As discriminative capacity of each epitope was detected, the selected epitopes were validated using human serum samples collected from school students exposed to an outbreak of toxoplasmosis [23] and from mother/newborn pairs. Finally, a larger group of human and cat sera confirmed to have *Toxoplasma gondii* anti-IgG antibody were serotyped using validated peptide-ELISA.

## Methods

### Sequencing of GRA6 and GRA7 genes

For the amplification of GRA6 and GRA7 genes, *T. gondii* strains that were previously isolated from stray cats were used [3]. Among these strains, 19 were type II, two were type III and one was of Africa 1 genotype in which type III and Africa 1 strains are rather rare for the region. Moreover, after complete genome sequencing of Africa 1 it is accepted as an individual lineage (without recombination) (L. Galal personal communication). During PCR, GRA6 and GRA7 genes were amplified as previously described with minor modifications using 5-GTAGCGTGCTTGTTGGCGAC-3 and 5-TACAAGACATAGAGTGCCCC-3 primers and 5-ACCCTATATTGGGGCTTGCT-3 and 5-ACACTGTCCTCGAGCTCCTA-3 primers [20, 21]. For each amplification reaction, 25 µl final volume contained 2 µl template DNA, 12.5 µl master mix (DreamTaq, Thermofisher), 1 µl from each primer (10 pmol/µl), and 8.5 µl distilled water. PCR was conducted using the following conditions: one cycle of 15 min at 95 °C for initial denaturation, 35 cycles of 94 °C for 30 s, 65 °C for 1.5 min, 72 °C for 1 min, and a final extension step at 60 °C for 30 min.

Purified PCR amplicons were sequenced by ABI Prism 3100 genetic analyzer. Sequences generated after translation into amino acid sequences by MEGA7 software were aligned with type II (Beverley; AAF60335.1) and type III (NED; AAF60337.1) reference sequences for GRA6 and type II (Beverley; ABV82436.1) and type III (NED; ABE69205.1) for GRA7. In addition, all detected polymorphisms were compared to the NCBI sequence databases to analyze whether they were novel.

### Prediction of B cell epitopes of GRA6 and GRA7 proteins

Linear B cell epitopes belonging to GRA6 and GRA7 proteins were predicted by Bcepred taking into consideration hydrophilicity, flexibility/mobility, accessibility, polarity, exposed surface and turns properties (https://webs.iiitd.edu.in/raghava/bcepred/bcepred_submission.html) [24]. Genotype specific epitopes were identified among predicted B cell epitopes which locate in the C-terminus of the GRA6 and GRA7 proteins and show properties of hydrophilicity, accessibility, flexibility and exposed surface or combinations of them [19, 25, 26]. In addition, the antigenicity value of each genotype specific B cell epitope was also analyzed by VaxiJen 2.0 (http://www.ddg-pharmfac.net/vaxijen/VaxiJen/VaxiJen.html). Table 1 shows epitope sequences, antigenicity value and epitope properties. Peptides harboring genotype specific epitopes were synthesized by Elabscience^®^ (Elabscience Biotechnology, Inc., Wuhan, China).

**Table 1:** GRA6 polymorphisms detected in *T. gondii* strains isolated from domestic cats

Genotype	Polymorphisms														
	7	47	50	105	180	185	198	205–210	211	217	219	223	224	227	230
Type II	Y	G	R	A	Q	G	G	–	E	R	P	G	S	E	F
Type III	H	D	K	V	P	D	R	YGGRGE	G	R	A	E	R	V	Y
Africa 1	H	G	Q	V	P	D	G	YRGRGE	G	G	A	E	R	V	Y
Beverley (type II)	Y	G	R	A	Q	G	G	–	E	R	P	G	S	E	F
NED (type III)	H	D	K	V	P	D	R	YGGRGE	G	R	A	E	R	V	Y

Polymorphism profile given for each genotype was detected in all isolates within genotype

### Docking analysis

3-D structure of each epitope was constructed by I-TASSER Server (http://zhanglab.ccmb.med.umich.edu/I-TASSER) and Protein Data Bank (PDB; http://www.rcsb.org/pdb/) was used to retrieve the 3-D structure of heavy and light chains belonging to the variable region of B cell receptor (PDB number: 5DRW). Each epitope was docked to B cell receptor by ClusPro Server (https://cluspro.bu.edu/home.php) and visualized on UCSF Chimera 1.14 tool.

### Serum samples

Serum samples (n:11) collected from cats naturally infected with type II, type III or Africa 1 genotype and confirmed to be anti-*T. gondii* IgG positive by a commercial ID.VET ELISA kit (ID Screen^®^ Toxoplasmosis Indirect Multi-species, France) [3] were used for the detection of discriminative capacity of each epitope synthesized. The ID.VET ELISA kit was also used for the confirmation of negative group cat serum samples (n:9). Human sera (n:30) collected from school students exposed to an outbreak of toxoplasmosis as well as from mother/newborn pairs (n:3) were used for validation of serotyping epitopes. In addition, serum samples obtained from humans (n:38) and cats (n:24) confirmed to have anti-*T. gondii* IgG antibodies in our previous studies were used to reveal the serotype profile of humans and cats. During peptide-ELISA, 18 human as well as nine cat seronegative sera were used as negative control to determine the cut off value.

### Peptide-ELISA

Peptide-ELISA was performed as described by Doel [27]. Briefly, each well of the plate (Nunc^®^, Denmark) was coated by peptide which was diluted to 2 µg/well in distilled water and incubated overnight at 37 °C until all distilled water evaporated. The well was blocked with a 1×PBS containing 0.5% BSA (v/w) for 1 h at room temperature and then washed three times with PBS-T [1×PBS containing 0.1% Tween 20 (v/v)]. The sera belonging to cats or humans were diluted to 1/33 in 1×PBS containing 0.5% BSA (v/w) and 0.1% Tween 20 (v/v) and incubated for 2 h at room temperature. The wells were washed thrice with PBS-T. Anti-human IgG antibody with HRP (Sigma, Germany) or anti-Feline IgG antibody (Invitrogen-Thermo Fisher) was diluted 1/5.000 in PBS-T and incubated for 40 min at room temperature. The wells were washed thrice with PBS-T and bound antibodies were visualized after adding 3, 3′, 5, 5′ tetramethylbenzidine (TMB) substrate. The reaction was stopped by adding 75 µl of 2 N sulfuric acid and the results were evaluated in a micro titer plate reader (Bio-Tek ELx808™, USA) at 450 nm. The cut off values of ELISAs were calculated using the receiver operating characteristic (ROC) analysis.

## Results

### Polymorphisms

Among *T. gondii* isolates from lineages type II, III and Africa 1 genotype, polymorphisms in GRA6 protein were detected at 20 different amino acid positions (Table 1). An insertion with six amino acids in length (YGGRGE) that is found in type III and Africa 1 isolates was remarkable. Also, polymorphisms in GRA7 protein were detected at 23 different amino acid positions (Table 2). Among these polymorphisms, valine and glutamine alterations found at positions 6 and 37 in type II genotype as well as phenylalanine alteration found at position 60 in type II and III were detected for the first time.

**Table 2:** GRA7 polymorphisms detected in *T. gondii* strains isolated from domestic cats

Genotypes	Polymorphisms																						
	6	8	37	60	106	115	120	160	161	167	170	172	176	182	185	194	199	201	202	220	222	229	231
Type II	I	F	**Q**	V	R	N	H	I	L	Q	T	E	T	S	L	A	M	L	T	L	Q	K	G
Type II	**V**	F	R	**F**	R	N	H	I	L	Q	T	E	T	S	L	A	M	L	T	L	Q	K	G
Type III	I	F	R	V	G	H	N	I	L	Q	T	D	S	S	I	V	L	I	K	P	H	E	R
Type III	I	F	R	**F**	G	H	N	I	L	Q	T	D	S	S	I	V	L	I	K	P	H	E	R
Africa 1	I	S	R	V	R	N	H	L	V	E	R	D	T	G	L	A	M	L	T	L	Q	E	G
Beverley (Type II)	I	F	R	V	V	N	H	I	L	Q	T	E	T	V	L	A	M	L	T	L	Q	K	G
NED (Type III)	I	F	R	V	G	H	H	I	L	Q	T	D	S	V	I	V	L	I	K	P	H	E	R

Novel polymorphisms are shown in bold

V, Q and F polymorphisms at positions 6, 37 and 60 were detected in two different isolates within type II whereas F polymorphism at position 60 was detected in one isolate within type III. Also, other polymorphisms given for each genotype was detected in all isolates within genotype

### Genotype specific B cell epitopes and design of peptides

A total of 22 B cell epitopes specific to each of the three lineages were predicted using Bcepred. Twelve of them were derived from GRA6 protein while the remaining 10 of them belong to GRA7 protein. Among GRA6 epitopes, five were specific to type II, five were specific to type III and the remaining two were specific to Africa 1 lineage. Of the GRA7 epitopes, four were specific to type II, four were specific to type III and the remaining two were specific to Africa 1 lineage. All epitopes were predicted to have high antigenicity value ranging from 0.8714 to 3.0947 by VaxiJen 2.0. Similarly, all epitopes were predicted to have properties of hydrophilicity and accessibility whereas most of them also had properties of flexibility and/or exposed surface. Detailed information about selected epitopes is presented in Table 3. Also, as the number of amino acid of an epitope was smaller than 10, it was synthesized as two repeat peptide.

**Table 3:** Peptide sequences, antigenicity value, and properties of selected peptides

Protein	Genotype	Epitope code	Peptide sequences	VaxiJen 2.0	Bcepred properties			
				Antigenicity value	Hydrophilicity	Exposed surface	Flexibility	Accessibility
GRA 6	Type II	GRA6II/a	CSPQEPSGGGSPQEPSGGG	1.4197 (Probable ANTIGEN)	+	+	+	+
		GRA6II/b	CSPQEPSGGG**GGSGGGSGSG**SPQEPSGGG	2.3756 (Probable ANTIGEN)	+	+	+	+
		GRA6II/c	CNNAGNGGNEGRG	2.6731 (Probable ANTIGEN)	+	−	+	+
		GRA6II/d	CPQEPSGGG**GGSGGGSGSG**NNAGNGG	2.7601 (Probable ANTIGEN)	+/+	±	+/+	+/+
		GRA6II/e	CGGNEGRG**GGSGGGSGSG**EGGEDDRRP	3.0947 (Probable ANTIGEN)	+/+	−/+	+/+	+/+
	Type III	GRA6III/a	CSPPEPSGDSPPEPSGD	1.4057 (Probable ANTIGEN)	+	+	+	+
		GRA6III/b	CSPPEPSGD**GGSGGGSGSG**SPPEPSGD	2.3851 (Probable ANTIGEN)	+/+	+/+	+/+	+/+
		GRA6III/c	CGNRGNEGRGYGGRGEG	2.7613 (Probable ANTIGEN)	+	−	+	+
		GRA6III/d	CYGGRGEGGGEDDRRA	2.6499 (Probable ANTIGEN)	+	−	+	+
		GRA6III/c	CRGNEGRGYGGRGEGGGEDDRRA	2.7573 (Probable ANTIGEN)	+	−	+	+
	Africa 1	GRA6 Africa 1/a	CGGNEGRGYRGRGEGGGEDDG	2.5140 (Probable ANTIGEN)	+	−	+	+
		GRA6 Africa 1/b	CYRGRGEG**GGSGGGSGSG**CYRGRGEG	2.6984 (Probable ANTIGEN)	+/+	−/−	+/+	+/+
GRA 7	Type II	GRA7II/a	CEQEVPESGKDGEDARQ	1.5594 (Probable ANTIGEN)	+	−	+	+
		GRA7II/b	CQEVPESGKDGQEVPESGKDG	1.5759 (Probable ANTIGEN)	+	−	+	+
		GRA7II/c	CLEQEVPESGKDGLEQEVPESGKDG	1.0891 (Probable ANTIGEN)	+	−	+	+
		GRA7II/d	CGLTRT**GGSGGGSGSG**PALEQEVPESGKDG	1.3801 (Probable ANTIGEN)	+/+	−/−	−/+	+/+
	Type III	GRA7III/a	CHEVPESGEDREDARQ	1.0538 (Probable ANTIGEN)	+	+	+	+
		GRA7III/b	CHEVPESGEDREDHEVPESGEDRED	0.8714 (Probable ANTIGEN)	+	+	+	+
		GRA7III/c	CPEHEVPESGEDR	0.9809 (Probable ANTIGEN)	+	+	+	+
		GRA7III/d	CGIKRT**GGSGGGSGSG**PAPEHEVPESGEDR	1.0359 (Probable ANTIGEN)	+/+	−/+	−/+	+/+
	Africa 1	GRA7 Africa 1/a	CEDGEDARQ**GGSGGGSGSG**EDGEDARQ	2.7831 (Probable ANTIGEN)	+/+	+/+	+/+	+/+
		GRA7 Africa 1/b	CEDGEDARQEDGEDARQ	1.5400 (Probable ANTIGEN)	+	+	+	+

Bold amino acid sequences indicate the linker sequence used to link two epitopes

+ shows that epitope has the tested property while − shows that epitope have not the tested property

± or −/+ indicate epitopes containing two linked epitopes which have different properties

### Docking

**Fig. 1:**
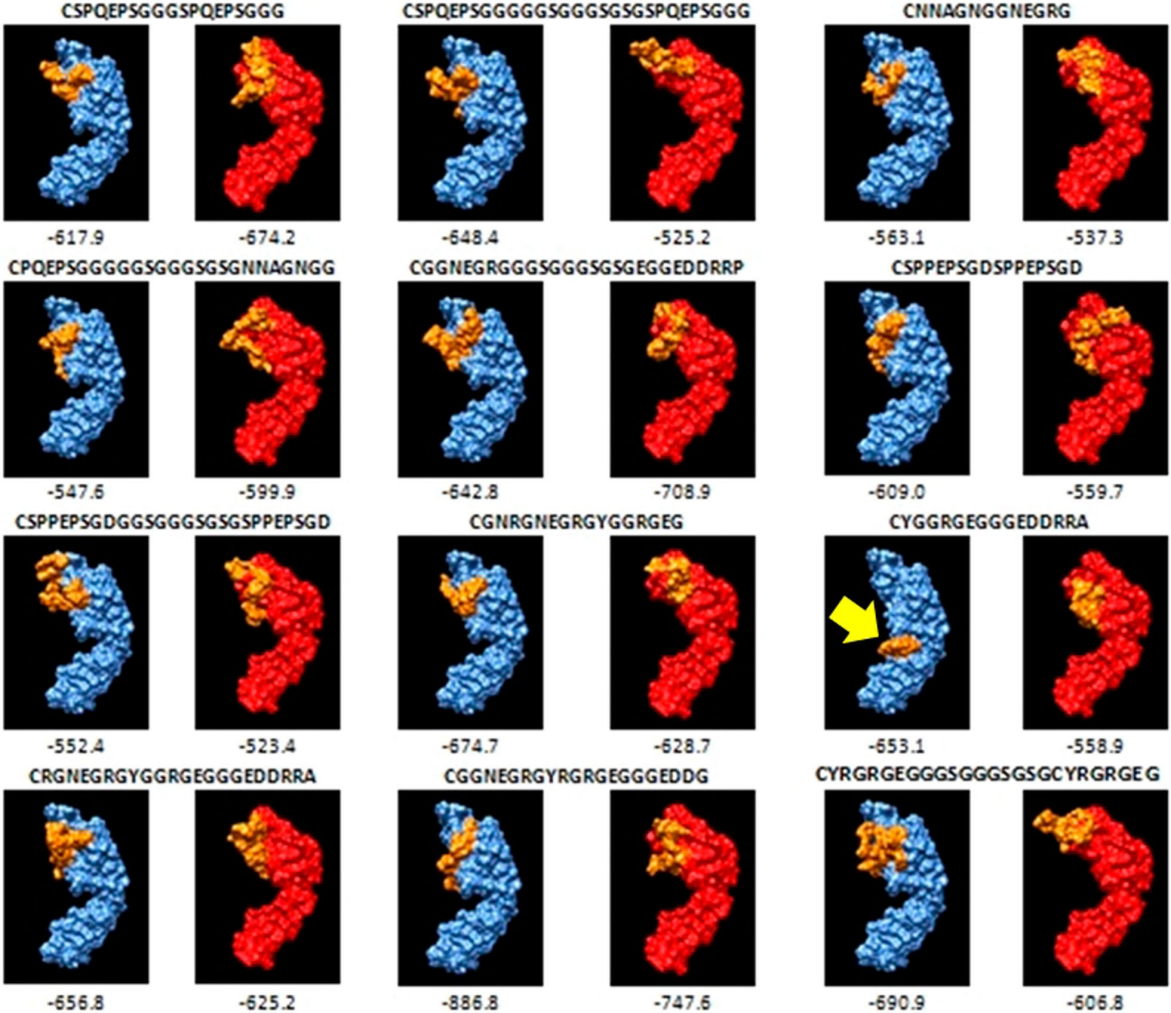
Docking results of epitopes derived from GRA 6 protein with variable region of heavy chain of B cell receptor. Blue color represents heavy chain; Red color represents light chain; Yellow color represents epitope. Yellow arrow shows epitope that was not correctly dock to variable region of heavy chain of B cell receptor

**Fig. 2:**
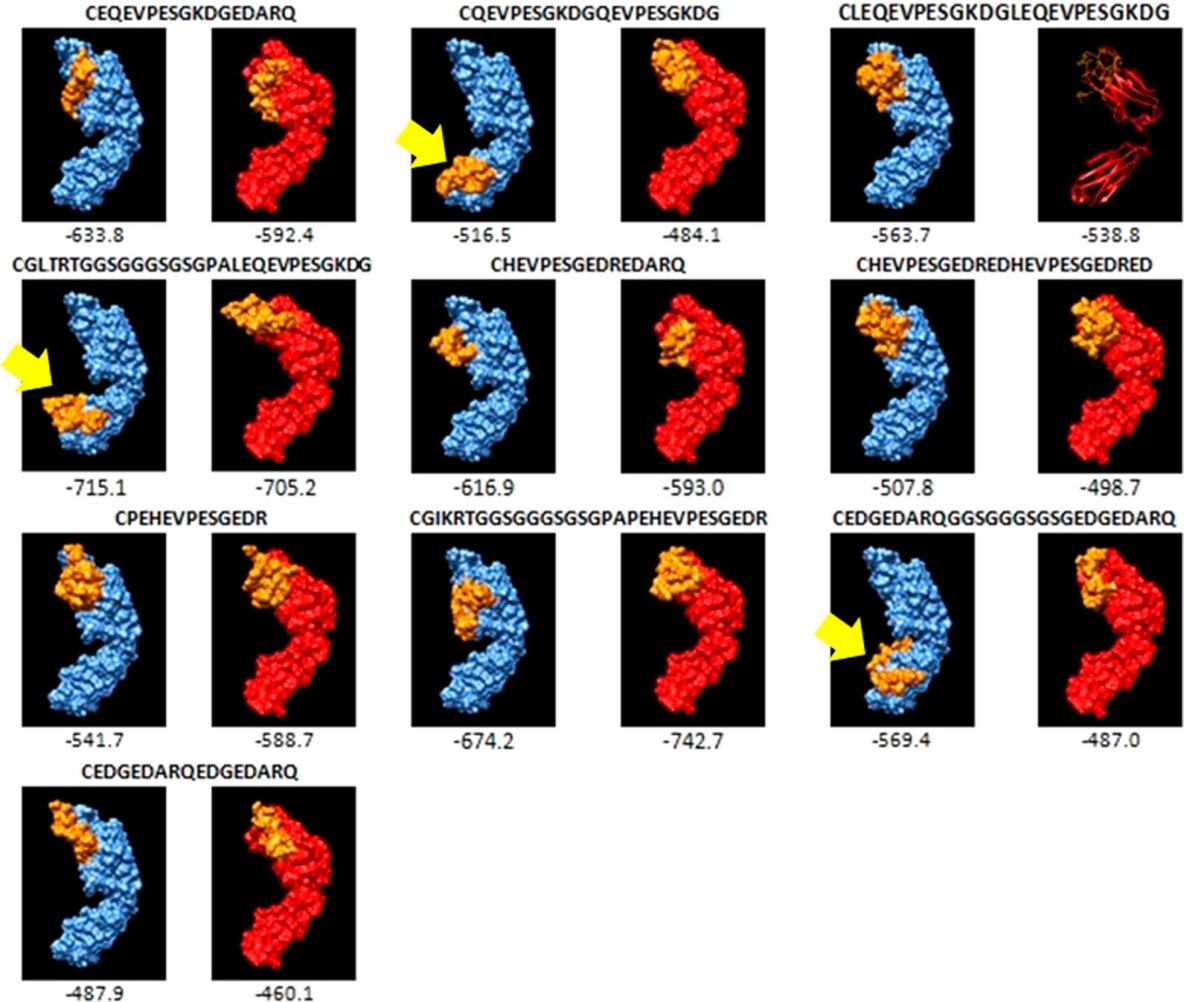
Docking results of epitopes derived from GRA 7 protein with variable region of heavy chain of B cell receptor. Blue color represents heavy chain; Red color represents light chain; Yellow color represents epitope. Yellow arrow shows epitope that was not correctly docked to variable region of heavy chain of B cell receptor

All selected epitopes were docked to the variable region of the light chain of B cell receptor with a high binding energy ranging from − 460.1 to − 742.7 whereas some epitopes (CYGGRGEGGGEDDRRA; CQEVPESGKDGQEVPESGKDG; CGLTRTGGSGGGSGSGPALEQEVPESGKDG and CEDGEDARQGGSGGGSGSGEDGEDARQ) were not correctly dock to the variable region of the heavy chain of B cell receptor although they had high binding energy (Figs. 1 and 2).

### Serotyping

**Fig. 3:**
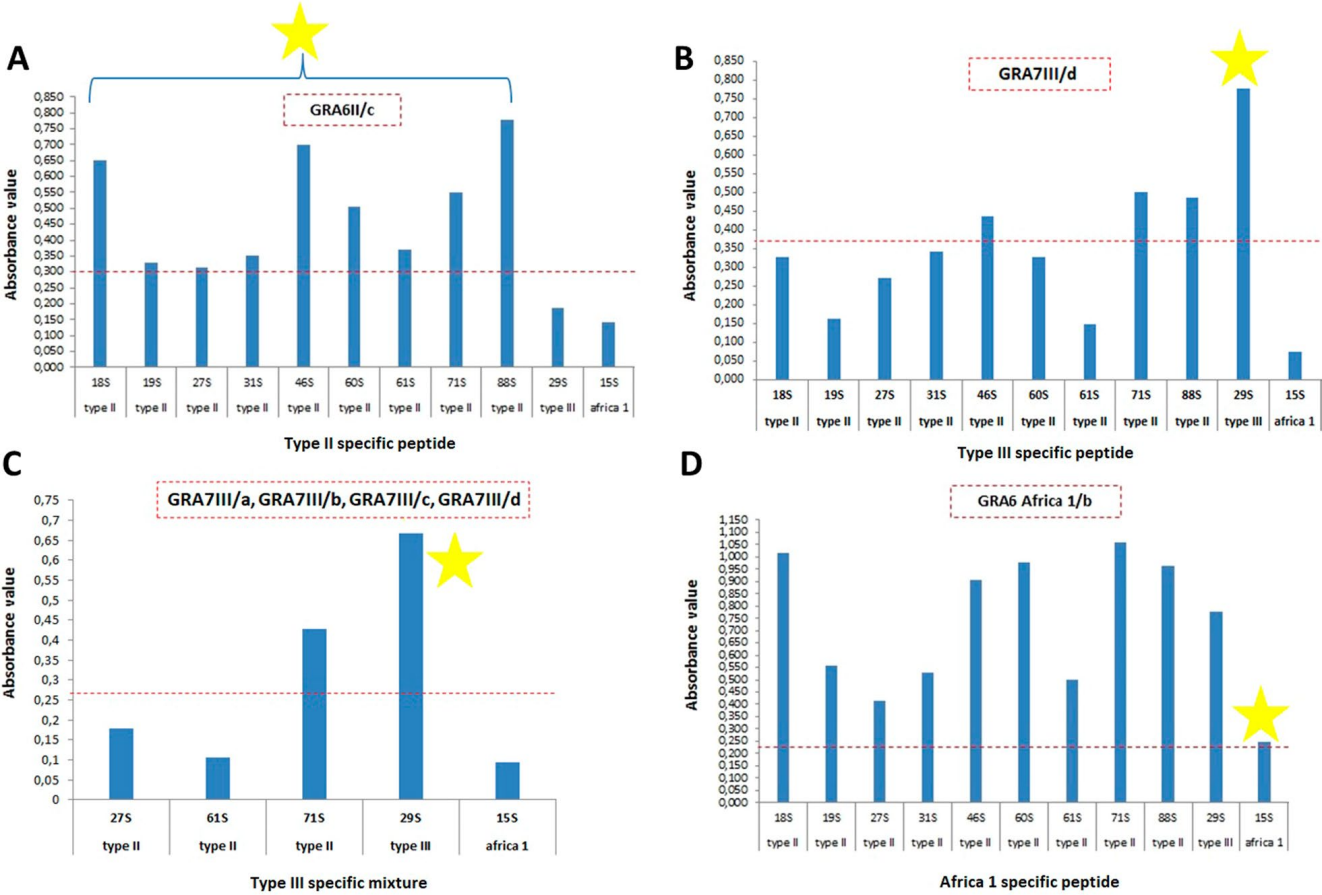
Peptide-ELISA results showing the serotyping capacity. Cut off value was calculated by ROC analysis using negative cat serum samples (n:9). The significant results obtained from each epitope are shown by star. Horizontal dotted lines show cut off values

Discriminative capacity of each peptide was analyzed by peptide-ELISA using well categorized sera obtained from domestic cats naturally infected with genotypes belonging to type II, III and Africa 1 lineages. Initially, all serum samples were probed by all epitopes to detect the discriminative capacity of each epitope as well as sensitivity and specificity values (Table 4). Among 22 epitopes, GRA6II/c epitope derived from GRA 6 protein reacted with all serum samples from cats infected with type II while no reaction was occurred with sera from cats infected with type III or Africa 1 genotype (Fig. 3-A). As all the remaining epitopes related to type II did not react with all cat sera infected with type II genotype, only GRA6II/c peptide was accepted as a marker for serotype II due to high discrimination capacity. Similarly, GRA7III/d peptide derived from GRA 7 protein reacted strongly with serum from cat infected with type III but at the same time it also reacted weakly with two serum samples collected from cats infected with type II (Fig. 3-B). In order to increase the serotyping capacity of GRA7III/d peptide, type III specific antigen mixture containing GRA7III/a, GRA7III/b, GRA7III/c and GRA7III/d peptides was used in peptide-ELISA. Type III peptide mixture also reacted strongly with sera of cat infected with type III. However, the mixture also reacted weakly with sera from cats infected with type II (Fig. 3-C). Any other type III related peptide, except GRA7III/d, didn’t react with sera of cat infected with type III. Interestingly, GRA7 Africa 1/a epitope reacted strongly with serum samples from cat infected with type III and type II, but did not reacted with serum from a cat infected with Africa 1 genotype. Another Africa 1 specific epitope “GRA6 Africa 1/b” reacted with serum sample collected from a cat infected with Africa 1 genotype. Also, this peptide showed the cross-reactivity among different genotypes by reacting with all serum samples from cats infected with type II and III (Fig. 3-D; Table 4).

**Table 4:** Sensitivity and specificity values of each peptide for the detection of *T. gondii* serotypes

Protein	Genotype	Epitope code	Sensitivity (%)	Specificity (%)	Cut off value	Cross-reactivity
GRA 6	Type II	GRA6II/a	55.5	100	0.32	–
		GRA6II/b	66.6	100	0.25	–
		GRA6II/c	100	100	0.3	–
		GRA6II/d	66.6	50	0.37	Type III
		GRA6II/e	55.5	100	0.35	–
	Type III	GRA6III/a	0	50	0.41	Type II
		GRA6III/b	0	40	0.28	Type II
		GRA6III/c	0	10	0.32	Type II
		GRA6III/d	0	40	0.30	Type II
		GRA6III/c	0	50	0.39	Type II
	Africa 1	GRA6 Africa 1/a	0	30	0.34	Type II
		GRA6 Africa 1/b	100	0	0.22	Type II and III
GRA 7	Type II	GRA7II/a	66.6	100	0.37	–
		GRA7II/b	44.4	100	0.37	–
		GRA7II/c	55.5	100	0.37	–
		GRA7II/d	33.3	100	0.26	–
	Type III	GRA7III/a	0	60	0.4	Type II
		GRA7III/b	0	60	0.38	Type II
		GRA7III/c	0	50	0.36	Type II
		GRA7III/d	100	70	0.37	Type II
	Africa 1	GRA7 Africa 1/a	0	40	0.37	Type II and III
		GRA7 Africa 1/b	0	60	0.34	Type II

– means no cross-reactivity was detected. Sensitivity and specificity values were obtained from serum samples collected from cats infected with type II (n:9), type III (n:1) and Africa 1 (n:1) genotype

**Fig. 4:**
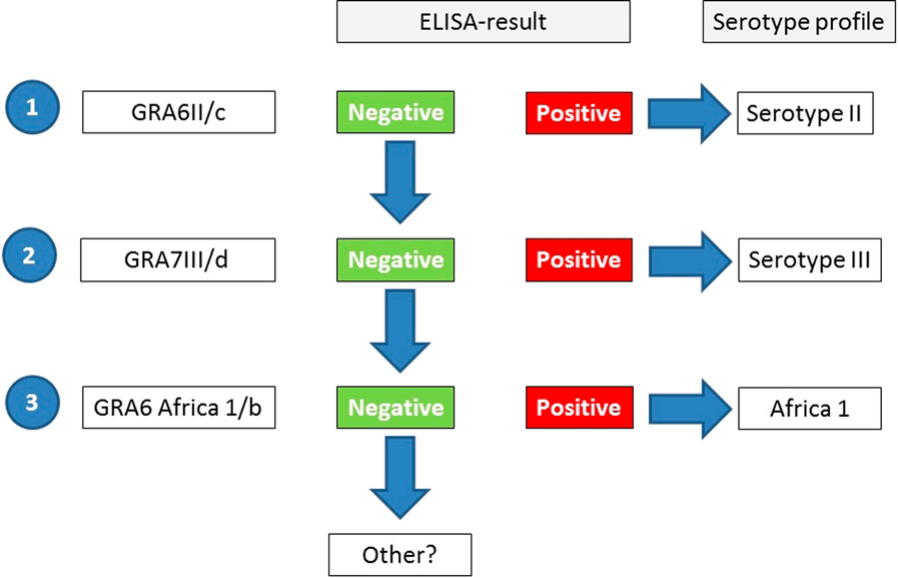
Simple illustration of serotyping schema

These findings obtained from analyses of cat sera demonstrated that utilization of GRA6II/c, GRA7III/d, and GRA6 Africa 1/b peptides enabled serotyping of serum samples infected with *T. gondii* and paved a way for the construction of a serotyping schema (Fig. 4). According to this schema, if a serum sample containing anti-*T. gondii* IgG antibody is found positive by GRA6II/c, it can be accepted as serotype II because this peptide only reacted with sera from cat infected with type II. On the other hand, if a serum sample is found negative by GRA6II/c, it should be studied by GRA7III/d and, if it reacts with GRA7III/d, it can be accepted as serotype III because this peptide was single peptide that reacts with serum sample from cat infected with type III. Finally, a serum sample that is found negative by both GRA6II/c and GRA7III/d should be studied by GRA6 Africa 1/b epitope and if it is found positive, this serum can be accepted as Africa 1 genotype or, if negative, it can be considered as either related to a strain from a lineage different from the first three lineages (type II, type III and Africa 1) or related to a different recombinant or atypical strain. This serotyping schema was used during validation and serotyping of the human and cat sera which were categorized in our previous studies [3, 28, 29].

### Validation of the serotyping peptides

Validation of the serotyping schema which is composed of GRA6II/c, GRA7III/d, and GRA6 Africa 1/b was used to determine the serotype responsible for a toxoplasmosis outbreak and in mother/newborn pairs. When these human serum samples were tested by GRA6II/c, GRA7III/d, and GRA6 Africa 1/b epitopes, all of them reacted with GRA6II/c. We therefore concluded that this toxoplasmosis outbreak was linked to a serotype II strain. All human sera collected during outbreak reacted with GRA6 Africa 1/b however four serum samples (patient 10, 13, 21 and 28) had an AV that is very close to the cut off value (Table 5). Additional validation was performed by serum samples with mother/newborn pairs (n:3) and among these serum pairs, all of them reacted with GRA6II/c and thus, all cases were accepted to be caused by serotype II. Also, all serum samples reacted with GRA6 Africa 1/b epitope as expected (Table 6).

**Table 5:** ELISA results belonging to serum samples collected after an outbreak of human toxoplasmosis

Patients	Peptides			Serotype
	GRA6II/c Cut off: 0.30	GRA7III/d Cut off: 0.16	GRA6 Africa 1/b Cut off: 0.18	
Patient 1	0.512	0.287	0.322	Serotype II
Patient 2	0.384	0.274	0.207	Serotype II
Patient 3	0.572	0.341	0.286	Serotype II
Patient 4	0.439	0.352	0.265	Serotype II
Patient 5	0.388	0.423	0.445	Serotype II
Patient 6	0.597	0.453	0.294	Serotype II
Patient 7	0.491	0.382	0.269	Serotype II
Patient 8	0.503	0.358	0.241	Serotype II
Patient 9	0.627	0.431	0.352	Serotype II
Patient 10	0.32	0.245	0.196	Serotype II
Patient 11	0.362	0.266	0.208	Serotype II
Patient 12	0.366	0.175	0.213	Serotype II
Patient 13	0.32	0.163	0.197	Serotype II
Patient 14	0.331	0.166	0.205	Serotype II
Patient 15	0.417	0.182	0.304	Serotype II
Patient 16	0.32	0.198	0.206	Serotype II
Patient 17	0.666	0.466	0.339	Serotype II
Patient 18	0.435	0.145	0.239	Serotype II
Patient 19	0.451	0.198	0.249	Serotype II
Patient 20	0.32	0.182	0.434	Serotype II
Patient 21	0.361	0.213	0.192	Serotype II
Patient 22	0.571	0.378	0.346	Serotype II
Patient 23	0.403	0.235	0.252	Serotype II
Patient 24	0.49	0.261	0.228	Serotype II
Patient 25	0.608	0.437	0.342	Serotype II
Patient 26	0.594	0.403	0.304	Serotype II
Patient 27	0.573	0.356	0.316	Serotype II
Patient 28	0.44	0.486	0.193	Serotype II
Patient 29	0.615	0.53	0.333	Serotype II
Patient 30	0.555	0.349	0.297	Serotype II

Cut off value was calculated by ROC analysis using negative human serum samples (n:18)

**Table 6:** ELISA results belonging to serum samples collected from mother/newborn pairs

Patients	Peptides			Serotype
	GRA6II/c Cut off: 0.16	GRA7III/d Cut off: 0.1	GRA6 Africa 1/b Cut off: 0.17	
Newborn	0.213	0.164	0.215	Serotype II
Mother	0.254	0.138	0.231	Serotype II
Newborn	0.358	0.255	0.383	Serotype II
Mother	0.249	0.096	0.238	Serotype II
Newborn	0.205	0.122	0.173	Serotype II
Mother	0.189	0.106	0.239	Serotype II

Cut off value was calculated by ROC analysis using negative human serum samples (n:18)

### Serotype profile of humans and cats

Serum samples collected from humans (n:38) and cats (n:24) with anti-*T. gondii* IgG antibodies were used to reveal the serotype profile in humans and cats. Of the 38 human sera, 37 (97.3%) were successfully serotyped using the validated peptide-ELISA approach. Unexpectedly, one serum sample belonging to patient 3 did not react with any epitopes and it was concluded that the human could be infected either with a strain from a lineage different from the three previous lineages type II, type III and Africa 1, either with a recombinant or atypical different strain.

Among human serum samples, serotype II was highly prevalent when compared to type III and Africa 1 serotypes. The prevalences of serotype II, serotype III and Africa 1 were therefore 86.8% (33/38), 7.9% (3/38) and 2.6% (1/38), respectively (Table 7).

**Table 7:** Serotyping results belonging to serum samples collected from human with toxoplasmosis

Patients	Peptides			
	GRA6II/c Cut off: 0.17	GRA7III/d Cut off: 0.12	GRA6 Africa 1/b Cut off: 0.17	Serotype
Patient 1	0.305	0.118	0.313	Serotype II
Patient 2	0.216	0.068	0.043	Serotype II
Patient 3	0.154	0.035	0.164	Other?
Patient 4	0.365	0.182	0.235	Serotype II
Patient 5	0.251	0.099	0.252	Serotype II
Patient 6	0.575	0.397	0.334	Serotype II
Patient 7	0.276	0.078	0.289	Serotype II
Patient 8	0.153	0.128	− 0.004	Serotype III
Patient 9	0.46	0.199	0.462	Serotype II
Patient 10	0.185	0.075	0.202	Serotype II
Patient 11	0.241	0.106	0.296	Serotype II
Patient 12	0.382	0.169	0.255	Serotype II
Patient 13	0.329	0.101	0.195	Serotype II
Patient 14	0.31	0.085	0.191	Serotype II
Patient 15	0.399	0.192	0.353	Serotype II
Patient 16	0.388	0.171	0.189	Serotype II
Patient 17	0.386	0.18	0.482	Serotype II
Patient 18	0.158	0.198	0.157	Serotype III
Patient 19	0.124	0.195	0.201	Serotype III
Patient 20	0.238	0.113	0.201	Serotype II
Patient 21	0.232	0.062	0.155	Serotype II
Patient 22	0.139	0.019	0.236	Africa 1
Patient 23	0.287	0.146	0.231	Serotype II
Patient 24	0.255	0.112	0.239	Serotype II
Patient 25	0.267	0.149	0.254	Serotype II
Patient 26	0.335	0.174	0.319	Serotype II
Patient 27	0.208	0.082	0.190	Serotype II
Patient 28	0.403	0.153	0.245	Serotype II
Patient 29	0.365	0.145	0.248	Serotype II
Patient 30	0.187	0.032	0.139	Serotype II
Patient 31	0.305	0.082	0.348	Serotype II
Patient 32	0.242	0.12	0.191	Serotype II
Patient 33	0.243	0.523	0.240	Serotype II
Patient 34	0.298	0.246	0.360	Serotype II
Patient 35	0.368	0.245	0.313	Serotype II
Patient 36	0.314	0.149	0.285	Serotype II
Patient 37	0.273	0.284	0.302	Serotype II
Patient 38	0.274	0.121	0.240	Serotype II

Cut off value was calculated by ROC analysis using negative human serum samples (n:18)

Serotype profile was also investigated in serum samples collected from cats. According to obtained results, all serum samples (100%) were successfully serotyped using the validated peptide-ELISA approach. Among serotyped serum samples, 23 of them (95.8%) were serotype II and the remaining one (4.16%) were serotype III (Table 8).

**Table 8:** Serotyping results belonging to serum samples collected from cats with toxoplasmosis

Number of cats	Peptides			
	GRA6II/c Cut off: 0.20	GRA7III/d Cut off: 0.15	GRA6 Africa 1/b Cut off: 0.22	Serotype
Cat 1	0.395	0.636	0.811	Serotype II
Cat 2	0.603	0.379	0.358	Serotype II
Cat 3	0.381	0.154	1.029	Serotype II
Cat 4	0.347	0.246	0.161	Serotype II
Cat 5	0.768	0.418	0.654	Serotype II
Cat 6	0.217	0.143	0.152	Serotype II
Cat 7	0.674	0.435	0.543	Serotype II
Cat 8	0.466	0.172	0.313	Serotype II
Cat 9	1.235	0.931	0.423	Serotype II
Cat 10	0.779	0.563	0.847	Serotype II
Cat 11	0.539	0.423	0.248	Serotype II
Cat 12	0.397	0.277	0.525	Serotype II
Cat 13	0.73	0.34	0.246	Serotype II
Cat 14	0.676	0.321	0.838	Serotype II
Cat 15	0.291	0.178	0.443	Serotype II
Cat 16	0.395	0.462	0.091	Serotype II
Cat 17	0.127	0.226	0.187	Serotype III
Cat 18	0.898	0.573	0.817	Serotype II
Cat 19	0.503	0.402	0.363	Serotype II
Cat 20	1.215	0.892	0.284	Serotype II
Cat 21	0.31	0.22	0.173	Serotype II
Cat 22	0.244	0.234	0.132	Serotype II
Cat 23	0.454	0.373	0.426	Serotype II
Cat 24	1.651	1.327	0.439	Serotype II

Cut off value was calculated by ROC analysis using negative cat serum samples (n:9)

Based on optimization and validation results that we expected, 38 human sera and 24 cat sera should have given reaction with GRA6 Africa 1/b epitope but six of human sera and six of cat sera did not give reaction with GRA6 Africa 1/b epitope despite they were serotyped as serotype II or III.

## Discussion

The parasite genotype and severity of clinical outcome in human toxoplasmosis cases are strongly related and in mouse model, type I is known to be more virulent compared to type II and type III [6]. It is recommended to type *T. gondii* isolates in clinical cases to assess the effect of genotype on the severity of toxoplasmosis [30, 31]. However, available routine genotyping methods that are based on the detection of parasite DNA are not always adequate because the amount of parasite DNA present in the host/patient’s fluids or tissues is not necessarily present at detectable or sufficient levels for genotyping and subsequent analysis. In addition, some samples that could have a higher parasite load and allow genotyping are not possible due to their invasive nature (heart, brain, aqueous humor…). Instead of parasite DNA detection, serological typing has become an alternative method with an easier biological sampling, and based on the detection of host antibodies rising against highly polymorphic parasite proteins among different genotypes [1, 18, 19].

To use this approach, preliminary bioinformatics analyses are needed. One of them is the prediction of B cell epitope regions on relevant antigenic protein and then the determination of the genotype-specific regions among them. In the selection of B cell epitope regions having some physico-chemical properties such as hydrophilicity, flexibility, accessibility and exposed surface as well as antigenicity are important parameters. The other preliminary bioinformatics analysis is docking which demonstrates potential interactions between predicted epitopes and the B cell receptor and supports obtained results [32, 33]. Before the synthesis of peptides, the present study applied this in silico approach for the selection of B cell epitopes specific to type II, III and Africa 1 lineages which represent the genetic diversity of *T. gondii* strains previously described in Turkey especially in cats and humans in Turkey [3, 4, 8]. For this, GRA6 and GRA7 genes were sequenced and B cell epitopes were predicted. Accordingly, a total of 22 B cell epitopes derived from GRA6 or GRA7 proteins were selected based on their lineage-specific properties and having hydrophilic, accessible, flexibility and antigenic assets as well as location in exposed surface (Table 3). Also, all epitopes were shown to dock to B cell receptor (Figs. 1 and 2).

According to peptide-ELISA results, a serotyping schema was constructed using GRA6II/c, GRA7III/d, and GRA6 Africa 1/b epitopes to serotype type II, III and Africa 1 genotypes. GRA6II/c discriminating the type II has sequences of “NNAGNGGNEGRG” and locates among amino acid positions 193 and 204 in GRA 6 protein. When the sequence was compared between type II and type III, it was found that there was a single amino acid difference caused by glycine (G) to arginine (R) alteration at position 198. Consistent with our study, a small part (NEGRG) of the GRA6II/c epitope was used in a previous study for the detection of type II [19]. This sequence together with continuing sequences was also used to detect the type I/III and atypical strain [19, 21]. Since the GRA6II/c epitope did not react with sera from cats infected with type III and Africa 1 genotype but reacted with all sera from cats infected with type II, sensitivity and specificity values of this epitope in detection of type II infection were accepted as 100% (Fig. 3A; Table 4). Based on this result, our serotyping schema strongly recommends analyzing serum samples with GRA6II/c initially to decide whether *T. gondii* infection is associated with serotype II or not.

Another promising epitope of this study, with GRA7III/d, had a sequence of “GIKRTGGSGGGSGSGPAPEHEVPESGEDR” and was made up of two different epitopes which are linked with a linker having sequences of “GGSGGGSGSG”. The first part (GIKRT) of GRA7III/d epitope has never been used before in previous studies, while part of the second part (PAPEHEVPESGEDR) of GRA7III/d has already been used for detection of type III [1, 19, 21]. The GRA7III/d epitope which is selected for the detection of type III reacted strongly with sera from cat naturally infected with type III as expected. However, the GRA7III/d epitope also reacted with three of nine sera from cats infected with type II (Fig. 3B). Also, when other type III related epitopes were added in addition to GRA7III/d epitope in order to solve cross-reactivity problem, results did not alter. This result shows that sensitivity of this epitope is higher than its specificity due to cross-reactivity occurring with type II (Table 4). The second recommendation of schema was that the serum samples that are found negative by GRA6II/c epitope need to be studied by GRA7III/d epitope to decide whether *T. gondii* infection is associated with serotype III or not.

The third promising epitope, GRA6 Africa 1/b, having sequences of “CYRGRGEGGGSGGGSGSGCYRGRGEG” is derived from an insertion sequences which is found in type III and Africa 1 genotype but not in type II. This epitope also differ between type III and Africa 1 genotype because of Glycine (G) to Arginine (R) alteration at position 206. A part (YRGRGEG) of the GRA6 Africa 1/b was used to serotype atypical strains in a previous study [19]. GRA6 Africa 1/b reacted with serum sample from cat infected with Africa 1 genotype in addition to other all sera from cats infected with type II and type III. GRA6 Africa 1/b was the single epitope reacting with serum sample from cat infected with Africa 1 genotype. This finding was important in two aspects. First, GRA6 Africa 1/b in combination with the previous two epitopes provides a diagnostic opportunity for toxoplasmosis in relation to a lineage that is rare for the region, but more importantly, a lineage that is potentially responsible for more severe forms of toxoplasmosis in the immunocompetent [16]. In addition, GRA6 Africa 1/b epitope, alone, has the potential to be used more widely in the diagnosis of toxoplasmosis because of reacting with most of serum samples tested. Additional tests are required to understand the diagnostic capacity of the GRA6 Africa 1/b epitope using a larger number of Africa 1 lineage sera to confirm our results and to test this epitope with other sera in relation to new lineages and atypical and/or recombinant strains. Finally, the third recommendation of the schema was that serum samples that are found negative by GRA6II/c and GRA7III/d epitopes are required to be studied by GRA6 Africa 1/b to decide whether *T. gondii* infection is associated with Africa 1.

In this study, validation of the serotyping schema constructed was performed by serum samples collected from an outbreak of human toxoplasmosis and mother/newborn pairs. According to the serotyping schema, all outbreak sera were serotyped as serotype II and this result was found to be compatible with the hypothesis that all patients were infected from a single source. All serum samples belonging to mother/newborn pairs were serotyped as serotype II and this was also compatible with the reality that mother and baby were infected with the same strain. These results together with those of the epidemic also confirm the predominance of the type II lineage in Turkey and more generally in Europe. The strength of this study was the validation of a novel peptide ELISA using sera obtained from a human outbreak and paired mother/newborn sera. On the other hand, the weakness of this study was the use of small number of serum samples collected from cats infected with type III and Africa 1 strains during validation experiments.

Type II strains have been predominantly isolated from humans and animals in Europe and North America [6, 34, 35]. Especially, Ajzenberg et al. [35] reported that congenital toxoplasmosis cases were predominantly caused by type II in Europe. Another study showed that type II strains were prevalent in immunocompromised patients who acquired toxoplasmosis in Europe [36]. In Turkey, two different studies conducted by our group reported that type II was more prevalent in stray cats whereas Africa 1 genotype was detected in two different congenital toxoplasmosis cases [3, 8]. In this study, the serotype profile of human serum samples was compatible with previous serotyping studies conducted in Europe because of detecting serotype II in high frequency (86.8%; 33/38) [37–39]. However, a single human serum could not be serotyped because of not reacting with any epitope whereas another serum that did not react with GRA6II/c and GRA7III/d epitopes and reacted with only GRA6 Africa 1/b epitope and thus serotyped as Africa 1 (Table 7). This situation can be explained by the possible presence of other lineages as well as different atypical or recombinant strains in the study area in addition to the three lineages tested here. It is therefore clear that additional epitopes are required for serotyping this potential greater diversity.

In this study, although serotype II was found as prevalent in stray cats (95.8%), type III also was found (4.16%). This result was in line with our previous study showing the high prevalence of type II in stray cats [3]. Besides, a study conducted in Germany reported that the prevalence of serotype II was very high in cats [22].

## Conclusion

In conclusion, this study demonstrates that simultaneous use of the three different epitopes including GRA6II/c, GRA7III/d, and GRA6 Africa 1/b is enough to serotype human or cat serum samples infected with all three lineages type II, III and Africa 1. Also, it was highlighted that additional epitope discovery is a requirement for serotyping of a potentially wider diversity. Finally, it was detected for the first time that serotype II was predominantly prevalent in humans and stray cats in Turkey despite the presence of other serotypes.

## Data Availability

All data generated or analyzed during this study are included in this article.

## Abbreviations

GRA: Dense granule protein
SAG: Surface antigen
BSR: Bradyzoite surface antigen
SRS: SAG1-related sequence
ROP: Rhoptry protein
NTPase: Nucleoside triphosphate hydrolase
IgG: Immune globulin G
AV: Absorbance value
SD: Standard deviation
DNA: Deoxyribonucleic acid
